# Sector Irradiation vs. Whole Brain Irradiation After Resection of Singular Brain Metastasis—A Prospective Randomized Monocentric Trial

**DOI:** 10.3389/fonc.2020.591884

**Published:** 2020-11-24

**Authors:** Johannes Kerschbaumer, Daniel Pinggera, Bernhard Holzner, Margarete Delazer, Thomas Bodner, Elfriede Karner, Lucie Dostal, Irma Kvitsaridze, Danijela Minasch, Claudius Thomé, Marcel Seiz-Rosenhagen, Meinhard Nevinny-Stickel, Christian F. Freyschlag

**Affiliations:** ^1^ Department of Neurosurgery, Medical University of Innsbruck, Innsbruck, Austria; ^2^ University Clinic for Psychiatry II, Medical University of Innsbruck, Innsbruck, Austria; ^3^ Department of Neurology, Medical University of Innsbruck, Innsbruck, Austria; ^4^ Department of Medical Statistics, Informatics and Health Economics, Medical University of Innsbruck, Innsbruck, Austria; ^5^ Department of Therapeutic Radiology and Oncology, Medical University of Innsbruck, Innsbruck, Austria; ^6^ Department of Neurosurgery, Klinikum Memmingen, Memmingen, Germany

**Keywords:** brain metastases, radiotherapy, stereotactic radiosurgery (SRS), quality of life, neuropsychology

## Abstract

To minimize recurrence following resection of a cerebral metastasis, whole-brain irradiation therapy (WBRT) has been established as the adjuvant standard of care. With prolonged overall survival in cancer patients, deleterious effects of WBRT gain relevance. Sector irradiation (SR) aims to spare uninvolved brain tissue by applying the irradiation to the resection cavity and the tumor bed. 40 were randomized to receive either WBRT (n = 18) or SR (n = 22) following resection of a singular brain metastasis. Local tumor control was satisfactory in both groups. Recurrence was observed earlier in the SR (median 3 months, 1–6) than in the WBRT cohort (median 8 months, 7–9) (HR, 0.63; 95% CI, 0.03–10.62). Seventeen patients experienced a distant intracranial recurrence. Most relapses (n = 15) occurred in the SR cohort, whereas only two patients in the WBRT group had new distant tumor manifestation (HR, 6.59; 95% CI, 1.71–11.49; p = 0.002). Median overall survival (OS) was 15.5 months (range: 1–61) with longer OS in the SR group (16 months, 1–61) than in the WBRT group (13 months, 3–52), without statistical significance (HR, 0.55; 95% CI, 0.69–3.64). Concerning neurocognition, patients in the SR group improved in the follow-up assessments, while this was not observed in the WBRT group. There were positive signals in terms of QOL within the SR group, but no significant differences in the global QLQ and QLQ-C30 summary scores were found. Our results indicate comparable efficacy of SR in terms of local control, with better maintenance of neurocognitive function. Unsurprisingly, more distant intracranial relapses occurred.

**Clinical Trial Registration:** ClinicalTrials.gov, identifier NCT01667640.

## Introduction

Surgical resection of intracerebral metastases leads to prolonged survival and relief of symptoms in selected patients (1). Solely local extirpation of the tumor mass does not solve the problem of local recurrence in the resection cavity, occurring in up to 50% of patients within the first year after operation (2).

Traditionally, whole-brain irradiation was the treatment of choice following surgical resection and has been the standard approach to minimizing the risk of intracranial recurrence following resection of brain metastasis (3). Almost two decades ago, Patchell et al. established the superiority of resection in patients with singular metastasis followed by whole brain irradiation as compared to standalone whole brain irradiation in terms of survival, local control, and maintenance of functional independence (4). A subsequent randomized trial by the same group, however, failed to show a survival advantage for additional whole brain irradiation as compared to surgical resection only in patients with a singular intracranial metastasis, although the likelihood of local and distant recurrence and death due to neurological causes was significantly reduced by whole brain irradiation (5). Due to potentially delayed neurocognitive effects associated with whole brain irradiation, investigators have evaluated the use of partial brain irradiation by means of stereotactic radiosurgery replacing whole brain irradiation after resection of brain metastases (6, 7). They showed that despite the fact that whole brain irradiation achieved superior control of distant brain recurrence, stereotactic radiosurgery after resection resulted in equivalent survival times and greater neurological preservation (8, 9).

This has also been highlighted in two recent phase 3 trials, where the addition of WBRT did not show any advantage in terms of prolonged overall survival (9, 10). Therefore, to preserve patients’ neurocognitive functions, a more localized treatment avoiding harm to the uninvolved brain is definitely warranted (11, 12).

The aim of this prospective randomized trial was to investigate whether postoperative, sector” - irradiation following surgical resection is equal to postoperative whole brain irradiation in terms of local control and superior in terms of quality of life and neurocognitive preservation.

## Materials and Methods

### Patients

Between April 2012 and April 2017, 40 patients were included in this prospective randomized controlled trial. To be eligible, patients had to present with singular brain metastasis (BM) with indication for surgical resection (tumor diameter >3cm, epilepsy or other symptoms refractory to medication, distinct patient’s wish). Also, patients had to present in good clinical condition defined as Karnofsky performance status >70 and stable extracranial disease. At the time of surgery for the BM, 27 patients were diagnosed as having systemic disease, but were staged in complete remission (CR) or in stable disease (SD). Only two patients showed progression at the site of primary manifestation with rational options in second-line systemic treatment. In the absence of progressive metastasis other than intracranial, these patients were enrolled. Patients who had previous cranial radiotherapy were excluded from the trial as well as patients with small-cell lung cancer (SCLC) and HER2-negative breast cancer.

Complete removal of the tumor on early postoperative MRI (within 72h after surgery) was mandatory to qualify for participation.

### Study Treatment

Every patient operated due to intracranial metastasis during the 5 years was screened for participation in this trial. If the inclusion criteria were fulfilled and the histology confirmed a BM, the opportunity to participate in the study was offered to the patients. Treatment was conducted at the discretion of the interdisciplinary tumor board.

**Figure 1:**
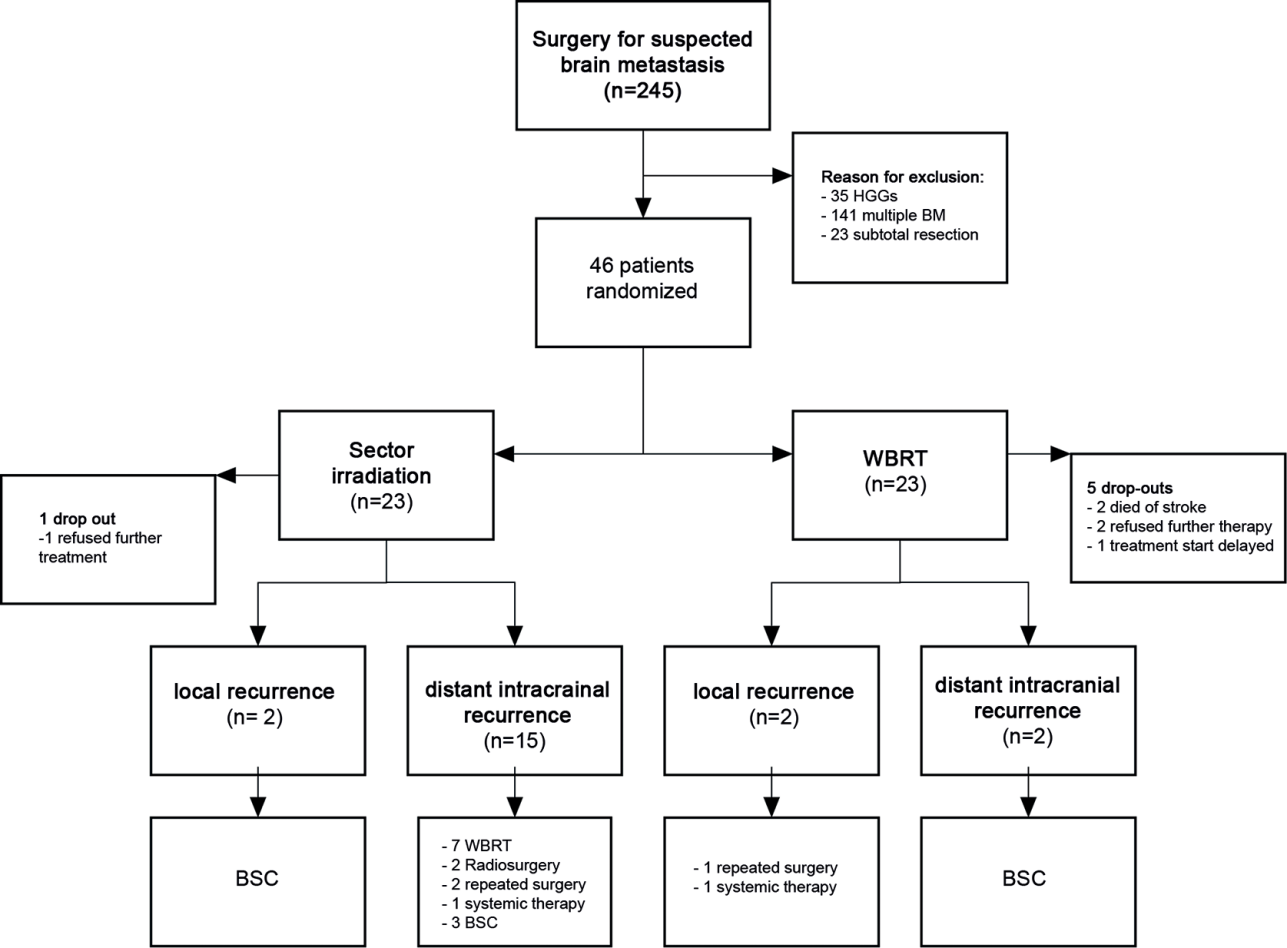
Treatment algorithm.

In the case of recurrence, salvage therapy was offered in terms of cross-over to WBRT for the patients in the treatment arm; repeated surgery or radiosurgical intervention was discussed individually in the case of intracranial progress (**Figure 1**).

### Neuroradiology

Standard cranial MRI as recommended for malignant brain tumors (13, 14) was performed within 72 h after resection to determine complete resection. The same standard cranial MRI sequences were performed every three months over the patients’ entire follow-up.

Progression was assessed using the RANO criteria for brain metastasis (15). In cases with suspected leptomeningeal spreading of the disease (LMD), presence of typical MRI features (ependymal spread, contrast enhancement of cranial nerves, cerebellar folia and cauda equina) was used to diagnose the presence of LMD.

### Histopathological Workup

Standardized histopathological diagnosis of the resected tissue was performed by HE staining and routine immunohistochemistry was used for further subtyping.

### Quality of Life Assessment

All patients underwent the MMSE as a screening measure of cognitive function. Executive functions were assessed with the EpiTrack battery (including the scores Interference, Trail making A and B, Labyrinth, Verbal fluency, Digit span backwards) and the Stroop Interference task. Verbal learning, verbal recall (short and long delay), and recognition of previously presented items were assessed with the VLMT (16–19).

“Patient-reported outcome” measurements were done prior to and at 3, 6, 12, and 24 months after radiotherapy. These included the EORTC QLQ-C30 questionnaire (version 3.0) and the BN20 module, both used frequently to assess neuro-oncological patients (20). Additional neuropsychological assessments were performed prior to and six, 12 and 18 months after radiotherapy by specialized neuro-psychologists blinded to the treatment arm.

### Study Randomization

After resection and histopathological confirmation, the patients were enrolled in the study and allocated to further treatment by 1:1 randomization before being subjected to radiotherapy. One group underwent standard WBRT, the other was treated with SR.

### Radiotherapy

Radiotherapy was administered within six weeks after surgery.

Patients were immobilized in a supine position using a frameless head mask fixation system (Brainlab AG, Munich, Germany) and simulated with a 1 to 1.25mm slice thickness computed-tomography scan.

Stereotactic planning computed tomography, planning MRI, and preoperative MRI datasets were imported to iPlan RT Image (v4.1.2, Brainlab AG, Munich, Germany) for image registration, fusion and contouring.

In the SR group, the gross tumor volume (GTV) was defined as the visible margin of the resection on post-operative MRI and planning CT scan. The clinical treatment volume (CTV) was the same as the GTV plus a 5mm margin (CTV = GTV + 5 mm). The planning treatment volume (PTV) had to include the CTV plus a 1mm margin in order to achieve preferably 95% of the volume at 95% of the dose. Additionally, organs at risk (OAR) would be delineated according to the ICRU 62 rules: brain stem, optic chiasm, both optic nerves, pituitary gland and both inner ears.

Dose prescription for the PTV was at or near the center of this volume following the recommendations of the ICRU 50/62 reports. Inhomogeneity correction for bone and soft tissue density variation was applied. The prescribed dose for the PTV was 30 Gray (Gy) in five fractions. Fraction size was 6 Gy, one fraction per day, five fractions per week.

Dose specification and homogeneity requirements in the PTV (−5%+7%) had to be in accordance with the ICRU guidelines. Optic chiasm, optic nerves and tracts should not receive a dose higher than 3Gy per fraction.

Administration was planned using the Pinnacle Treatment Planning system (versions 3 to 9, Philips Healthcare, Fitchburg, WI, USA). Isodose distributions were calculated through the target in three planes, transverse, coronal and sagittal. For each isocenter the following will be reported: number of arcs or fields, tangle angle, gantry start and stop angles, collimator settings, dose to target, dose to target for each arc, maximum dose. Isodose distributions in three planes with marked PTV and isodose lines with maximum dose, 90%, 80%, 60%, 50%, 40%, 20% of the prescription dose were reported. Dose volume histograms were reported.

In the WBRT arm the planning CT was be performed with 2 mm slice thickness. Fixation was done in a simple thermoplastic mask. The isocenter was defined in the frontal area of the brain in the middle of lamina cribrosa. The plan was a 3 D plan with lateral opposite reclined fields. The energy consists in 6MV or 15MV doses, depending on the anatomical shape of the brain. The prescribed dose was 40Gy on the 95%-Isodose, which means 20 fractions and a single dose of 2Gy.

Treatment was delivered with a linear accelerator with 6 MV photon energy Synergy S (ELEKTA Medical Intelligence Medizintechnik, Stockholm, Sweden). In room imaging was provided with a cone beam CT and I Guide positioning system (ELEKTA Medical Intelligence Medizintechnik, v2.2.2, Stockholm, Sweden).

### Statistical Analysis

Descriptive statistics, including median and ranges for continuous variables and counts and percentages for categorical values, were used to recapitulate the two groups.

As primary endpoint, both local and distant intracranial progression were defined analyzing MRI scans employing the RANO criteria (15).

For calculation of overall survival time to local and distal in-brain recurrence and time to systemic progression, the Kaplan-Meier method was used.

The trial was planned as non-inferiority study; power analysis was conducted considering time to progression as primary outcome. Herein a difference in time to local and distant progression of 3 months was rated clinically significant with an expected overall survival of 12 months mean. Thus, power calculation for sample size considered 36 patients sufficient to reach a power of 80%. To compensate for expected drop-outs, a total of 40 patients was included in the study.

Secondary endpoints were time to neurocognitive deterioration and quality of life, tested with a questionnaires described above.

For the neuropsychological assessments non-parametric tests were used (Mann-Whitney U test for between-group comparisons, Wilcoxon test for within-group comparisons).

Because of the small number of patients, a multivariate analysis was not performed. Therefore, a p value of <0.05 was considered statistically significant.

Due to expected drop outs, a per protocol analysis was conducted after 40 patients allocated.

Statistical analysis was performed using SPSS (IBM Statistics, v. 21, Armonk, NY, USA) and Prism (GraphPad, v. 6, La Jolla, CA, USA).

## Results

### Patient Characteristics

Forty-six patients with a singular BM were randomized in this prospective randomized study. Median age of the patients was 59 years (range: 34–79 years). Sex distribution was balanced between the two groups with a minor predominance of male gender in both groups. Mean time between primary diagnosis of systemic disease and the appearance of the central nervous system disease was 14 months (range: 0–55 months).

The most frequent systemic diagnosis was non-small cell lung cancer (NSCLC), involving more than half of the patients in the trial (n = 21, 52.5%).

Twelve patients received a diagnosis of systemic cancer at the time of cerebral manifestation (7 in the SR arm and 5 in the WBRT arm) (**Table 1**). As mentioned in the inclusion criteria, all patients presented with a good Karnofsky Performance Status (KPS>70) and showed a singular intracerebral metastasis.

**Table 1:** Patient characteristics.

	SR (n = 22)	WBRT (n = 18)	
**sex, n (%)**			
Male	13 (59)	10 (55)	*n.s.*
Female	9 (41)	8 (45)	*n.s.*
**Age**			
<50	2	2	*n.s.*
50–65	12	12	*n.s.*
>65	8	4	*n.s.*
**Primary**			
NSCLC^+^	11	10	*n.s.*
Melanoma	4	2	*n.s.*
Breast CA^+^	2	0	*n.s.*
Others*	5	6	*n.s.*
**Tumor size mm (min-max)**			
	33.4 (16–56)	27.6 (15–55)	
**Edema mm (min-max)**			
	67.3 (47–110)	58.3 (17–100)	
**Tumor location**			
Frontal	9	7	
Parietal	5	5	
Occipital	2	4	
Temporal	2	1	
Infratentorial	4	1	
**systemic @diagnosis**			
1st diagnosis	7	5	*n.s.*
CR*^+^*	12	8	*n.s.*
SD*^+^*	2	5	*n.s.*
PD*^+^*	1	1	*n.s.*

+ NSCLC, non-small-cell lung cancer; CA, cancer; CR, complete remission; SD, stable disease; PD, progressive disease.

*Others: in SR group, 2 ovarian CA; 1 prostate CA, 1 colon CA, 1 sarcoma in WBRT group: 2 CUP, 2 renal-cell CA, 1 ovarian CA, and 1 gastric CA.

Median follow-up of the 40 patients was 15.5 months (range: 1–61 months).

### Local Control

Local tumor control was satisfactory in both groups. There were two local relapses in each group. The recurrence occurred earlier in the SR group (median 3 months, 1–6) than in the WBRT cohort (median 8 months, 7–9) (HR, 0.63; 95% CI, 0.03–10.62) (**Table 2**).

**Table 2:** Outcome parameters.

	SECTOR (n = 22)	WBRT (n = 18)
Dead	11	12
Alive	11	6
**Overall survival**		
Mean	16	13
Min	1	3
Max	61	52
**Local relapse, months**		
n (%)	2	2
TTP*^+^*, median (range)	3(1–6)	8(7–9)
**Distant intracranial relapse, months**		
n (%)	15	2
TTP*^+^*, median (range)	6(1–39)	6(4–33)
**Systemic relapse, months**		** **
n (%)	8	9
TTP*^+^*, month (range)	5.5(0–12)	5(2–28)
**1st progression after treatment**		
Cerebral local	1	0
Cerebral distant	11	1
Systemic	4	10
**Rescue therapy**		
WBRT*^+^*	7	0
RS*^+^*	2	1
Surgery	2	1
Chemotherapy	1	0

^+^TTP, time to progression; WBRT, whole-brain radiotherapy; RS, radiosurgery.

### Distant Brain Control

In total, 17 patients experienced distant intracranial recurrence (**Table 2**) within a median time to distant recurrence of six months (range: 1–39 months). Unsurprisingly, most of the distant intracranial relapses occurred in the SR cohort (n = 15), whereas only two patients in the WBRT group had a new tumor manifestation at sites other than the surgical cavity (HR, 6.59; (95% CI, 1.71–11.49); p = 0.002).

### Leptomeningeal Disease

Leptomeningeal disease (LMD) was suspected in ten patients (25%) during the follow-up, occurring at a median time of eight months (range: 1–39 months). Only one LMD was proven in the WBRT cohort with typical spread to spinal nerve roots, but missing intracranial manifestation.

Four (10%) patients in the SR group and one patient in the WBRT cohort presented with typical MRI features of LMD; three of these patients were further assessed by means of CSF cytology and showed no evidence of tumor cells. Four (22%) patients in the SR cohort were suspected of having leptomeningeal spread due to new solid lesions (3–8) along the CSF pathway, but had no suspicious contrast-enhanced lining along the ventricular ependyma or contrast enhancement (CE) of the cranial nerves and had negative CSF cytology.

One patient in the SR group developed dural metastasis in the cervical spine 39 months after resection of a cerebellar metastasis without any further disease within the CNS. In the WBRT arm one patient developed a meningeal spread along the thoracic and lumbar spine.

Four of the patients who developed LMD were operated on a singular infratentorial BM, and three other patients presented with a BM including meningeal adherence.

Median overall survival after manifestation of LMD was four months (range: 0–16 months).

### Overall Survival

At the time of final assessment 23 (57.5%) patients had died; 13 (56%) of the patients died from progressive systemic disease without significant neurological burden. Seven (30.4%) patients deceased from the sequelae of neurological deterioration due to intracranial tumor progression. One patient died from a pulmonary embolism unrelated to treatment and two patients died of an unknown cause.

**Figure 2:**
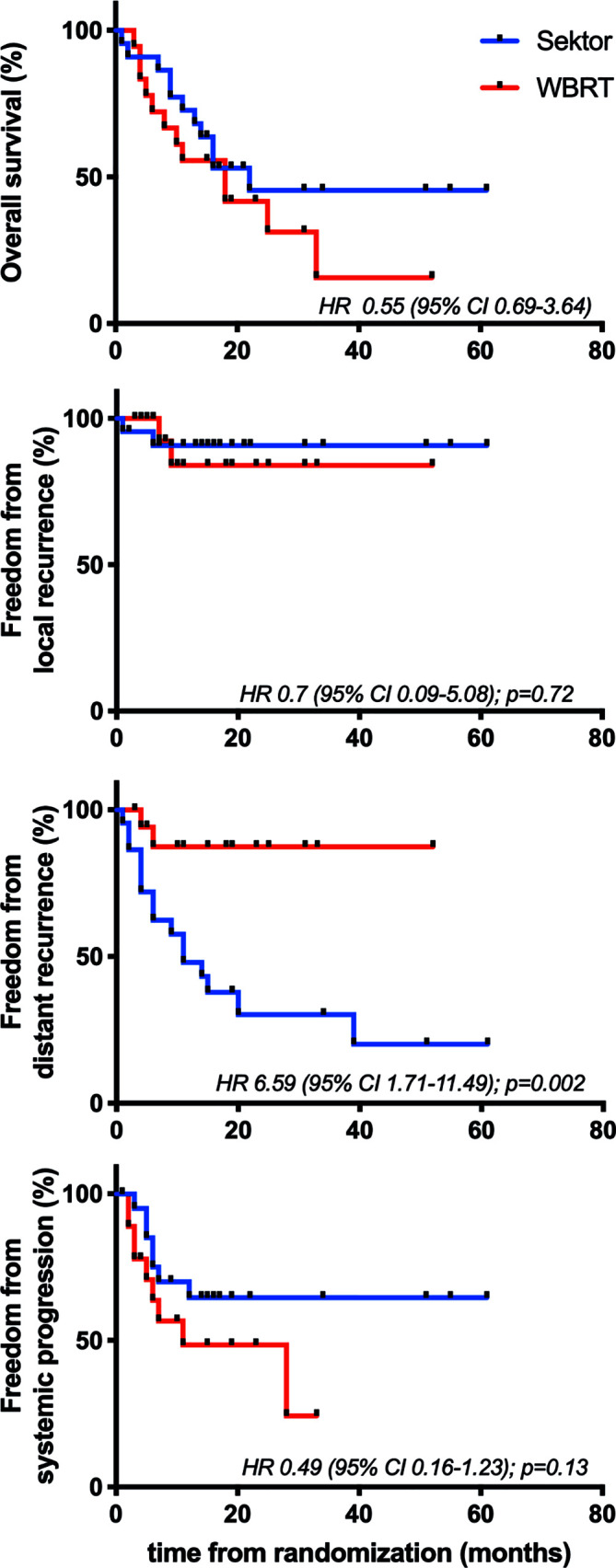
Kaplan-Meier estimates of overall survival, freedom form local and distant recurrence and systemic progression.

Analysis using the Kaplan-Meier method showed a median OS of 15.5 months (range: 1–61 months) with a slightly longer mean OS in the SR group (16 months, 1–61) than in the WBRT group (13 months, 3–52), but without statistical significance (HR, 0.55; 95% CI, 0.69–3.64) (**Figure 2**).

### Salvage Treatments

Two patients in the SR group with local recurrence in the resection cavity refused any further treatment and were assigned for best supportive care. Local relapses in the WBRT group were treated by repeated surgical resection in one case and systemic therapy in the other.

Seventeen patients developed distant brain recurrence during the follow-up. Two of them were already treated with WBRT after surgical resection. They presented in critical neurological deterioration and thus did not receive further treatment.

In the SR group 15 patients progressed by the mean of distant intracranial failure. Seven patients underwent additional WBRT after previous SR as the first therapeutic intervention, two patients with a single distant lesion were treated with radiosurgery, two patients required surgical excision of a distant BM and one patient was assigned for systemic treatment only. Three patients underwent no further treatment.

Patients with suspected LMD as first manifestation of progression were assigned to acute WBRT (n = 6); only one received surgery for a large spinal metastasis before irradiation of the spinal column. Two patients did not qualify for further treatment.

During the course of this trial, eight patients required WBRT after initial SR at a median time of nine months (range: 0–25 months).

### Quality of Life Assessment

For all 40 patients in the SR (n = 22) and the WBRT (n = 18) group baseline quality-of-life (QOL) data were available. Because of drop-outs in both groups, only 19 patients in the SR and 17 patients in the WBRT group were able to complete the test three months after randomization, qualifying for analysis in terms of deterioration due to the radiotherapeutic intervention. Only three patients in each group completed all six evaluation points (up to 24 months postoperatively).

There were positive signals in terms of better QOL within the SR group, especially concerning hair loss, which was a bigger problem in the WBRT group at three and six months after radiotherapy (p = 0.051 and p = 0.003). However, this between-group difference lost impact in further assessments. Also, patients in the SR group complained of fewer communication deficits at six and 18 months after radiotherapy (p = 0.032 and p = 0.048).

In contrast, patients in the WBRT group complained of less loss of appetite shortly after radiotherapy (p = 0.034), and drowsiness was also less frequent in the WBRT cohort three months after radiotherapy (p = 0.033).

**Figure 3:**
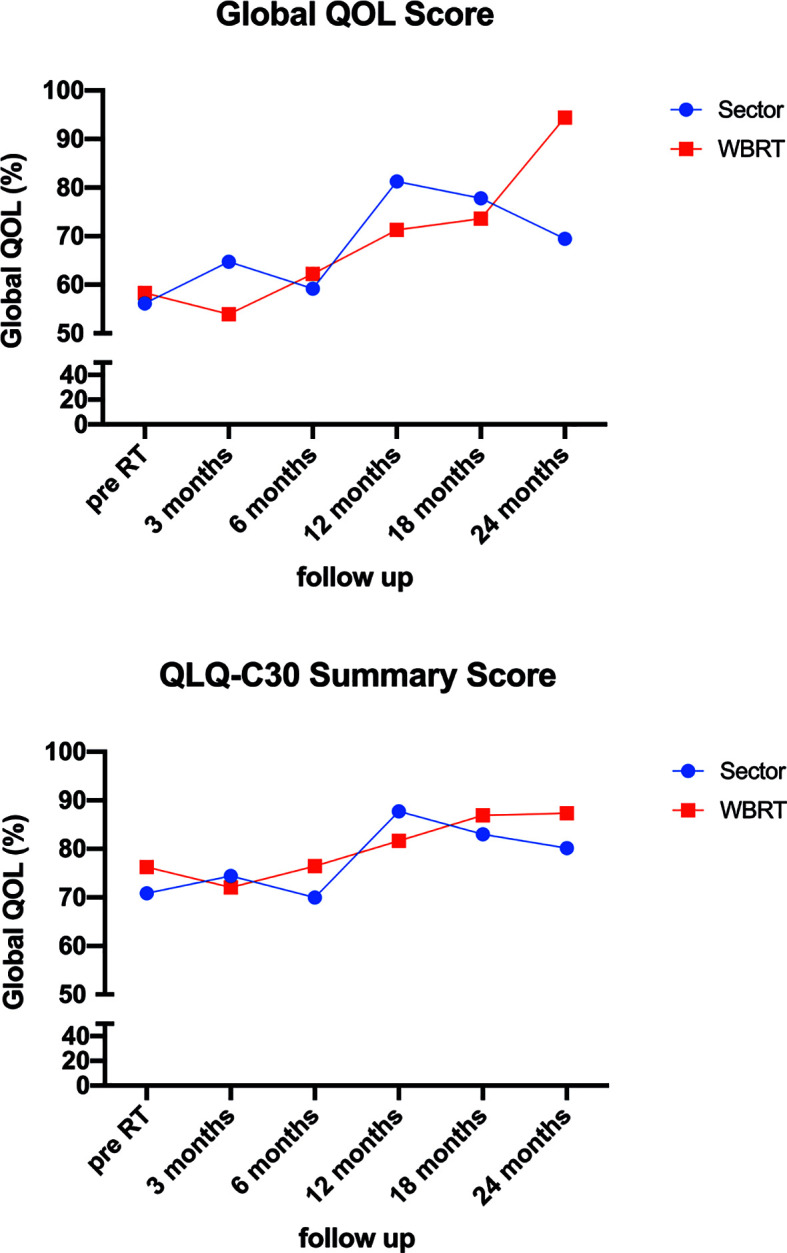
Quality of life assessment.

When combining the distinct sub-test and calculating the global QLQ and the QLQ-C30 summary score, no statistically significant differences were found (see **Figure 3**).

### Neuropsychological Assessment

The first assessment was completed by 19 patients in the SR group, while all 18 patients in the WBRT group were assessed prior to radiation therapy. Two assessments were available for comparison in eight patients after SR and in 11 patients after WBRT. Thereafter, fewer patients were available for analysis. Hence, a reliable statistical analysis could not be performed after this point (third assessment with 7/8 (SR/WBRT) patients, fourth with 4/5 patients and fifth with 1/1).

Eight patients (5 female) with SR radiation (SR) and 11 (2 female) with whole brain radiation (WBR) underwent two assessments. Patients did not differ in age. Education (years of formal education) was higher in the WBRT group.

Groups showed no significant difference in neuropsychological tasks (**Table 3**) in the first or second assessment. Comparisons between assessments (A1, A2) revealed that the SR group had higher MMSE scores in the second assessment than in the first (p = 0.026, Wilcoxon Test), while this was not the case for the WBRT group. Other comparisons did not achieve significance.

**Table 3:** Demographic and neuropsychological variables; between-group comparisons.

	Sector Irradiation (n = 8; 5 female)			Whole Brain Irradiation (n = 11; 2 female)			M.W.Tests p values
	Median	Perc 25^th^	Perc 75^th^	Median	Perc 25^th^	Perc 75^th^	
**Age**	57.5	51.0	65.5	56.0	51.0	63.0	n.s.
**First assessment**							
MMSE	26.5	25.0	28.0	28.0	26.0	29.0	n.s.
EpiTrack (sum score)	25.0	18.0	32.0	30.0	29.0	34.0	n.s.
Stroop Test Interference (sec.)	113.0	92.0	149.0	80.5	72.5	103.0	n.s.
Verbal memory VLMT							
Verbal learning(sum)	39.5	37.0	46.0	43.0	34.0	49.0	n.s.
Recall short delay (n)	7.0	6.0	12.0	8.0	6.0	9.0	n.s.
Recall long delay (n)	7.0	7.0	12.0	8.0	6.0	9.0	n.s.
Recognition (correct minus false answers)	10.5	8.0	15.0	11.0	8.0	12.0	n.s.
**Second assessment**							
MMSE	28.0	27.0	29.0	29.0	27.0	29.0	n.s.
EpiTrack (sum score)	25.0	17.0	30.0	30.0	19.0	32.0	n.s.
Stroop Test Interference (sec.)	110.5	85.5	123.5	97.0	85.0	101.0	n.s.
Verbal memory VLMT							
Verbal learning(sum)	42.0	35.5	47.5	44.0	33.0	47.0	n.s.
Recall short delay (n)	8.5	7.0	10.5	9.0	4.0	10.0	n.s.
Recall long delay (n)	8.5	6.5	10.0	7.0	3.0	10.0	n.s.
Recognition (correct minus false answers)	14.0	12.5	14.5	13.5	11.0		n.s.

## Discussion

In this single-center randomized controlled trial of patients with surgically resected, singular brain metastasis followed by either postoperative WBRT or SR, we found excellent local control (90%). Unsurprisingly, patients in the SR arm had a higher rate of distant in-brain relapse, even though this showed no statistically significant effect on overall survival.

Concerning quality of life, no signals favoring WBRT over SR were found, even if in the sub-scores for QLQ C30 and BN20 differences between the two groups were manifested.

To our knowledge, there are only few recent randomized controlled trials that investigated limited radiotherapeutic intervention after surgical resection of brain metastasis. Our trial aimed not only to investigate local tumor control, but also to evaluate neurocognitive outcome and quality of life.

Lately, it has become evident that WBRT after resection of a limited number of brain metastases, namely up to three lesions, showed increased control of the brain metastatic disease, but numerous large trials failed to show improved overall survival (3, 5, 9, 10). This fact was evident also in our series: we found comparable overall survival of 13 months in the WBRT arm and 16 months in the SR arm (HR, 0.55; 95% CI, 0.69–3.64).

A clear drawback of this trial is the quite high rate of distant intracranial failure in the SR arm, but this did not translate to reduced overall survival and is in line with results published for other studies (9, 11).

In general, leptomeningeal disease in systemic cancer becomes evident in 5%–15% of patients during the course of disease (21). However, many trials lack a clear definition of leptomeningeal disease, which makes comparisons between these trials challenging. Usually, CSF cytology, typical meningeal lining of the CE along the CSF pathways and the cranial nerves in the MRI or the clinical presentation are cited as criteria for diagnosing leptomeningeal spread.

Surgical resection of BMs has been named as a risk factor leading to tumor cell spread along the CSF pathway (22), and especially resection in the posterior fossa seems to favor leptomeningeal disease (23, 24). However, comparison of trials analyzing the rate of leptomeningeal disease after resection and subsequent WBRT (11%) (25) and sole radiosurgery for multiple intracranial metastases (13%) (24) permits no clear conclusion to be drawn on the true cause of surgical dissemination.

In our series, we found a crude rate of 25% leptomeningeal dissemination when applying a quite overestimating definition. Seven (17.5%) out of ten patients developed multiple (3-8) new metastases adjacent to the CSF pathway, but even in repeated CSF cytology revealed no clear CSF spread. One patient developed a single cervical metastasis after resection and SR of the posterior fossa that mandated repeated surgery and additional local radiotherapy.

Three patients developed spinal CE lining without cranial manifestation in the MRI scans.

Only two patients (5%) were rated as leptomeningeal spread due to classical CE lining of the cranial nerves and the ventricular ependyma. This may explain the high rate of LMD in our series when including all the cases that in comparable trials may be classified as multiple intracranial and intraspinal recurrences. Comparable radiosurgical treatments reveal a wide range of LMD rate, namely from 24% (26) to 8% (27).

Furthermore, increasing knowledge of negative long-term effects of WBRT on neurocognitive function (11, 12, 28) added to the need for alternative treatment for such patients. Contemporary targeted treatment enabled increased systemic tumor control. Therefore, neurocognitive decline became a clinically relevant issue. The knowledge that WBRT decreased the incidence of local or distant in-brain recurrence but did not affect overall survival in patients with BM brought on discussions of the value of WBRT among global neuro-oncology experts.

Many centers restricted WBRT for patients with oligometastatic intracranial disease in a palliative setting, especially in patients with a high risk for major neurological deterioration.

In this trial we elucidated the role of limited radiotherapy in a selected population with limited in-brain dissemination. By excluding patients with pre-existing cerebral treatments and selecting patients with a singular BM in the context of limited or stable systemic disease, we aimed to investigate the benefits of neurocognitive preservation and quality of life in our patients. The inclusion criteria were set to provide a cohort of patients with limited metastatic disease, in order to obtain medium to long-term results.

Decline rates in learning and memory function of up to 52% already within the first few months, as described in other series (12), clearly demonstrate the burden on patients treated with WBRT. These decline rates become increasingly important in patients with limited disease who will survive even longer.

In our study, patients with WBRT showed stable performance in the MMSE, while patients with SR improved their score in the second assessment (p = 0.026).

These results are in line with those of a recently published series that showed a deterioration in neurocognition in 85% of its patients within the first six months after WBRT (9). Surprisingly, we found no significant differences in the Epi track or the VLMT test battery to prove a reliable difference in neurocognitive salvage due to limited radiotherapy. Possibly, this finding may be due to the fact that fractionation with 2 Gy in 20 sessions also leads to better neurocognitive preservation in our patients undergoing WBRT. It is known that other centers apply higher fractions, namely 3 Gy per session, and it is possible that the higher single dose accounts for the poorer performance in the published WBRT series (9).

Clearly, the high rate of salvage WBRT in the case of multiple intracranial recurrences may equalize this rate also in patients with initial localized radiotherapy. This leads to additional neuropsychological decline in most patients in the same series described above. Eventually, however, this can prolong the time to deterioration in selected patients. In our series, the limited number of patients prevented a meaningful interpretation of these data. We thus excluded patients from neuropsychological assessment after progression and additional radiotherapy.

A clear shortcoming evident in this investigation is the quite small number of patients enrolled as compared to larger phase 3 trials in a multicenter setting. The rationale behind this cohort size was the trial’s intention to exclusively select patients with the best prognostic condition, e.g. singular BM, systemic well-controlled disease and complete resection of the CE tumor on early postoperative MRI. For this reason we were able to exclude many potential confounders, interfering with the oncological and neurocognitive outcome and directly investigating the benefits and negative impact of the conducted radiotherapeutic intervention.

Unfortunately, the small number of patients precludes a definitive answer addressing the long-term neurocognitive preservation, even if some positive signals were documented, at least in the short term. Of course, the result may also be tempered by the different location of the resected and irradiated BMs as we know that irradiation including of the hippocampus may lead to a greater decrease in neurocognitive function than does radiotherapy of regions distant to structures involved in memory function.

In patients with brain metastatic disease quality of life plays an important role, which is why we also assessed QLQ and QLQC30. It is also known and it has been reported that neurocognitive functions are linked to activities of daily life and thus may be correlated with quality of life. We were able to show differences in communication deficits for up to 18 months; these differences were significantly larger in the WBRT group. Even if the summary scores did not differ significantly, the subgroups may show the benefit of local radiotherapy methods.

## Conclusion

In this single-center prospective randomized trial we could prove that SR was efficient to maintain local control in selected patients after surgical resection of a single brain metastasis. Better maintenance of neurocognitive function could be shown measured by implementation of MMSE, even if distinct neurocognitive testing did not display significant differences, when compared with WBRT using 2 Gy fractions. Unsurprisingly, distal intracranial relapses occurred more frequently, which lead to more salvage therapies.

## Data Availability Statement

The raw data supporting the conclusions of this article will be made available by the authors, without undue reservation.

## Ethics Statement

The studies involving human participants were reviewed and approved by Ethics committee of the Medical University of Innsbruck. The patients/participants provided their written informed consent to participate in this study.

## Author Contributions

JK, DP, MS-R, CT and CF were responsible for the concept and conduction of the trial and drafted the presented manuscript. BH analyzed the QoL-assessments. MD, TB and EK were responsible for the neuropsychological assessment of the included patients. LD conducted the statistical analysis. IK, DM and MN-S were responsible for the conduction and evaluation of the irradiation technique. All authors contributed to the article and approved the submitted version.

## Conflict of Interest

The authors declare that the research was conducted in the absence of any commercial or financial relationships that could be construed as a potential conflict of interest.
